# Analysis of the sleep period and the amount of habitual snoring in individuals with sleep bruxism

**DOI:** 10.4317/medoral.23136

**Published:** 2019-10-27

**Authors:** Marcelo Palinkas, Julio Marrara, César Bataglion, Jaime Hallak, Graziela de Luca Canto, Priscilla Hakime Scalize, Isabela Regalo, Selma Siéssere, Simone Regalo

**Affiliations:** 1 DDS, PhD, Professor. Department of Basic and Oral Pathology, School of Dentistry of Ribeirão Preto, University of São Paulo; Faculty Anhanguera, Ribeirão Preto and National Institute and Technology - Translational Medicine (INCT.TM), São Paulo, Brazil; 2 DDS. Faculty of Dental Medicine, University of Porto, Portugal; 3 DDS, PhD, Professor. Department of Restorative Dentistry, School of Dentistry of Ribeirão Preto, University of São Paulo, São Paulo, Brazil; 4 DDS, PhD, Professor. National Institute and Technology - Translational Medicine (INCT.TM), São Paulo, Brazil; 5 DDS, PhD, Professor. Department of Dentistry, Federal University of Santa Catarina, Florianopolis, Brazil and Department of Dentistry, University of Alberta, Edmonton, Canada.; 6 DDS. Department of Basic and Oral Pathology, School of Dentistry of Ribeirão Preto, University of São Paulo, São Paulo, Brazil

## Abstract

**Background:**

The literature does not report any association between habitual snoring and sleep bruxism, but these situations can be a reason for frequent complaints of individuals, impairing the quality of life. This study was performed to investigate the sleep period and amount of habitual snoring in individuals with sleep bruxism observing expiratory, inspiratory, and mixed snoring.

**Material and Methods:**

A total of 90 individuals were screened and divided into the following groups: with sleep bruxism (n=45) and those without sleep bruxism (n=45). Single night sleep polysomnography was performed to diagnose sleep bruxism, quantify habitual snoring and sleep period. The results were tabulated and submitted to a Multivariate analysis of variance (MANOVA) to compare the means of the two independent groups, considering the affected diagnosis of sleep bruxism, snoring (independent variables) and age as covariate. For the post hoc, was used correcting for multiple comparisons (Bonferroni test, * P* <.05).

**Results:**

There was statistically significant difference among the groups (* p* =.001) in the sleep period, in that individuals with sleep bruxism slept for a longer duration (with sleep bruxism group: 460 minutes and without sleep bruxism group: 401 minutes). There were no statistically significant differences among the groups for the number of inspiratory, expiratory and mixed snores, but was observed greater amount of snoring in the with sleep bruxism group.

**Conclusions:**

The main finding of this study is that individuals with sleep bruxism slept longer than the control group. It may also be suggested that individuals with sleep bruxism tended to increase the amount of habitual snoring during sleep.

** Key words:**Bruxism, adult, sleep disordered breathing, polysomnography.

## Introduction

In 2013, bruxism was described as the repetitive, involuntary orofacial activity of the muscles that insert into the mandible. Moreover, bruxism was characterized by muscular rhythmic masticatory activity and was associated with clenching and grinding of the teeth during the mandibular tasks (1).

Nowadays this concept has changed. In healthy individuals, bruxism should not be considered as a disorder. It is a behavior that can be a risk or protective factor which could have clinical consequences (2). It presents with two circadian manifestations and can occur during sleep (sleep bruxism) or wakefulness (awake bruxism) (3). The etiology of bruxism is not well understood (4).

Sleep bruxism is considered a behavioral problem and has a prevalence rate of 8% of the adult population (5). The characteristic clinical signs include tooth wear, headache, and temporomandibular disorder (6,7).

Simultaneously with the orofacial consequences, some authors have suggested that sleep bruxism may be associated with psychological changes, such as stress, depression or anxiety, which through disturbances in the central nervous system, and with obstructive sleep apnea syndrome (8). However, the association between this behavior activity and the amount of habitual snoring produced during sleep has not yet been determined.

Snoring is a noise produced by the vibration of the soft parts of the aerodigestive tract walls (9). Snoring appears in 15–54% of the general population (10).

Snoring, which can be habitual, is defined according to the International Classification of Sleep Disorders as the presence of noise characteristic of snoring during sleep in the absence of alterations in oxyhemoglobin saturation, as determined by ventilatory measures and electroencephalography (11).

The main hypothesis of the study was that sleep bruxism does not change the amount of snoring during sleep, but lower sleep time. Therefore, this study aimed was to quantify the sleep period and amount of habitual snoring in individuals with sleep bruxism observing expiratory, inspiratory, and mixed snoring.

Materials and Methods

- Study design and subject

The present study was approved by the Research Ethics Committee (protocol no. 02735812.9.0000.5419), based on Resolution 466/2012 of the Brazilian National Health Council. Informed consent was obtained from all individual participants included in the study.

A post hoc was conducted considering a level of α=0.05, a power of 100% for the main outcome sleep time, sleep bruxism group = 460 [5] and without sleep bruxism group = 401 [6]), effect size of 11.68. The minimal sample size obtained was 90 participants (45 for each group). Sample size calculation was performed with the G*Power software v 3.0.10 (Franz Faul, Kiel University, Kiel, Germany).

Among the 280 individuals of the city of São Paulo, Brazil, both genders, ages between 18 and 45 years and with normal occlusion, 65 individuals were submitted a one single-night polysomnographic. The inclusion criteria were report of noise due to grinding teeth during sleep by the family; wearied facet; satisfactory overall health, and nasal breathing.

The individuals were excluded if they presented with a history of neurological and chronic degenerative diseases, hypothyroidism, temporomandibular disorders, and any objection to take the polysomnography test.

After polysomnography, 20 individuals were excluded from the sample for the following reasons: presence of obstructive sleep apnea syndrome (n = 8), unconfirmed diagnosis of sleep bruxism (n = 7) and use of systemic medication prior to polysomnography that could interfere with the results, such as muscle relaxants, anxiolytics, and antidepressants (n = 5). It total, 45 individuals with sleep bruxism were included in the case group.

Healthy individuals, without temporomandibular disorders, normal occlusion, were matched to the sleep bruxism group by age, gender, and body mass index (BMI) on a one-by-one basis. Individuals without sleep bruxism were also examined by polysomnography.

The two groups were distributed as follows: with sleep bruxism (29 men, 16 women; mean age: 30.5 ± 6.7 years; mean BMI: 25.6 ± 0.6 Kg/m2; n = 45) and without sleep bruxism (29 men, 16 women; mean age: 29.44 ± 7.8 years; mean BMI: 25.2 ± 0.6 Kg/m2; n = 45).

There were no significant differences (95% CI) between groups in terms of age (*p* = .98) and BMI (*p* = .22). Matching for factors such as age and BMI is convenient method for minimizing confounding in case-control studies, because balance the clinical characteristics of the two groups (12). The interview and clinical evaluation with consecutive individuals were performed by a single examiner, a trained dentist.

- Polysomnography (gold standard)

The individuals slept for one night in a sleep laboratory in Ribeirão Preto, São Paulo, Brazil and polysomnographic (PSG) recording were performed, by a qualified professional.

PSG was used to confirm sleep bruxism, rule out obstructive apnea sleep syndrome (apnea index > 5 per hour of sleep), quantify the number of habitual snores (expiratory, inspiratory, and mixed), and determine total sleep time.

The Meditron-Sonolab 620 device (Sao Paulo, Brazil), which has 26 A/C programmable channels, 6 DC constant channels, and low consumption and noise level, was used. It was coupled to 5000 v multi-user software, Windows XP/2000/NT, and 32-bit C++ compiler.

To confirm the diagnosis of sleep bruxism, the presence of more than four episodes of rhythmic masticatory muscular activity per hour of sleep, two or more teeth grinding episodes with noise per night, and more than 25 electromyogram bursts of sleep rhythmic masticatory muscle activity with SB per hour of sleep was required (13,14).

To record the number of expiratory, inspiratory, and mixed snores, a small-sized microphone with a frequency of 50–30,000 Hz was used. The microphone was attached to the subject's neck with 3M™ Micropore™ surgical tape (hypoallergenic). The high-frequency noise was removed with a low-pass filter (cut-off frequency: 200 Hz) and an Hsu's algorithm (15,16) was used to detect snoring and quantify the snoring events during sleep.

Individuals were informed that the polysomnography could be discontinued at any time. Factors inherent to the comfort of the individuals in the performance of polysomnography, such as pillow type, bed, ambient temperature, sounds and noises, and the fixing of electrodes to avoid discomfort, were considered.

- Statistical analysis

The study variables are presented as the mean ± standard deviation. The Kolmogorov-Smirnov test was used to evaluate the normality of the variables. The data were found to be normally distributed. The results were tabulated and submitted to a Multivariate analysis of variance (MANOVA) to compare the means of the two independent groups, considering the affected diagnosis of sleep bruxism, snoring (independent variables) and age as covariate. For the post hoc, was used correcting for multiple comparisons (Bonferroni test, *P*<.05). Student-t test for independent samples was used to compare age and BMI. The IBM SPSS Statistics for Windows, version 21.0 (IBM SPSS, IBM Corp., Armonk, NY, USA), was used for the statistical analysis.

## Results

Considering MANOVA findings, our results showed no main effect for the group (*Pi*llai´s trace= .02, F= 0.58, *p* = .67 [df=83]) and no effect for age (*Pi*llai´s trace= .02, F=0.55, *p*=.69 [df=83]). In addition, our results did not verify the interaction between group and affected age (*Pi*llai’s trace = 0.02, F = 0.65, *p* =.65 [df = 83]).

Considering such finding, we decided to describe the mean value for variables included in this study (Table 1). Individuals with sleep bruxism slept for a longer period (with sleep bruxism group: 460 minutes and without sleep bruxism group: 401 minutes). There were no statistically significant differences among the groups for the number of inspiratory (*p*=.06), expiratory (*p*=.28) and mixed (*p*=.07) snores, but there was trend for a greater amount of snoring in the SBG group.

**Table 1:**
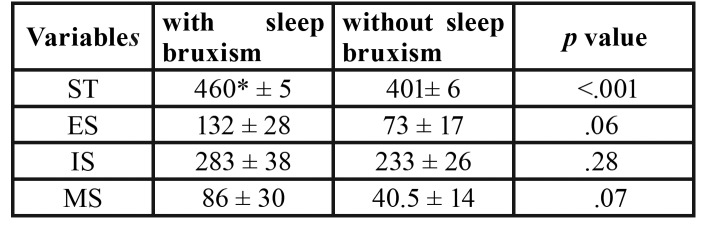
Mean (standard deviation) and statistical significance (*p*<.001) of sleep time (ST; minutes) and amount of expiratory (ES), inspirational (IS), and mixed (MS) snores for the with sleep bruxism (SBG) and without sleep bruxism (CG) groups. *Post hoc Bonferroni (*p*<.001).

## Discussion

Habitual snoring is a common disorder that arises at the onset of sleep, progressively increasing as sleep progresses to deeper stages. Only in the last few years has this condition been evaluated in detail by medical and dental researchers (17,18).

Considering the knowledge that snoring is often associated with obstructive sleep apnea syndrome (19), this study aimed to determine if sleep bruxism interfered with habitual snoring. These results are unprecedented in the international scientific literature due to the absence of studies that correlate sleep bruxism and snoring.

In the present study, it was observed that the group with sleep bruxism had a significantly longer sleep period than the control group. A probable explanation for this finding is in the amount of microarousals of the two groups (20) and the relation to the snoring.

We found that the group with sleep bruxism did not show a significant difference in the number of expiratory, inspiratory, or mixed snores compared with the control group, though a trend towards an increased number of snores was observed in the sleep bruxism group.

Anatomical changes during the respiratory process can provide valuable information in the evaluation of habitual snoring (21). The low-frequency noise of habitual snoring is produced by vibration of the uvula, soft palate, oropharyngeal region, tongue, and epiglottis, and is caused by increased airflow resistance in the upper airways (22). It is known that when there is sagging of the soft palate, for example, there is vibration during sleep, producing snoring (23). The impact of involuntary movements of the jaw during sleep on soft tissue sagging of the oral cavity and oropharyngeal region should be addressed in future research.

The hypothesis that involuntary movement of the jaw during sleep in individuals with sleep bruxism promotes floppy soft orofacial tissue, consequently increasing the amount of snoring, has not yet been studied and could provide valuable information.

Another hypothesis yet to be studied is the impact of anatomical position during sleep. Anatomical position, particularly the dorsal decubitus position, could increase the amount of snoring since the pharyngeal and tongue musculature may collapse against the posterior wall of the pharynx due to the loss of pharyngeal tissue (24). In the present study, sleep position was not evaluated during the polysomnographic examination.

It is known that sleep deprivation induces habitual snoring due to increased muscle relaxation, compromising contractions of the pharyngeal dilator muscles. These previous data are not consistent with the results of the present study, in which the group with sleep bruxism did not report sleep deprivation and was observed during polysomnography to have a longer sleep period than the group without bruxism. A longer sleep period does not necessarily equate to better sleep quality (20).

Several factors, such as the sporadic use of alcoholic beverages, smoking, or sedative medications, may worsen or increase the amount of snoring because of increased resistance in the airways (25-27). In this study, individuals with sleep bruxism did not use sedative medications; however, alcohol consumption and smoking were not assessed.

While snoring is considered normal in the general population, it can be a serious problem when great vibrations and intense noise is present. Such cases can lead to alterations in the quality of life for the individual as well as for bed partners and family members (28).

The results of this study led us to reflect on whether sleep bruxism may be associated with symptoms not expected by medical and dental professionals, such as inspiratory, expiratory, and mixed snoring, and demonstrate the need to evaluate this behavior in a morphological context.

We found that sleep bruxism can influence the amount of snoring and conclude that the results obtained explain the possible hypotheses. Further research should be conducted to support the results of this study and determine the precise association between sleep bruxism and increased inspiratory, expiratory, and mixed snoring.

These findings would allow for more precise diagnoses in the medical and dental areas. Additionally, such data would demonstrate to health professionals that the sleep bruxism might interfere with rehabilitative treatments, as the soft orofacial tissues might be more flaccid due to the intense and frequent involuntary mandibular movement, thereby resulting in a considerable amount of habitual snoring in individuals with sleep bruxism. The results of the present study should be interpreted with caution.

Some limitations of our study must be acknowledged. The assumed trend must be controlled for the sleep duration, as sleeping longer means a higher probability of more snoring per se. Sleep position, alcohol consumption and smoking are factors that could strongly influence snoring. The “single night effect” should also be taken into account.

## Conclusions

The main finding of this study is that individuals with sleep bruxism slept longer than the control group. It may also be suggested that individuals with sleep bruxism tended to increase the amount of habitual snoring during sleep. Further studies should be performed to support our findings.
